# Pharmacological Cardioversion after Pre-Treatment with Antiarrythmic Drugs Prior to Electrical Cardioversion in Persistent Atrial Fibrillation: Impact on Maintenance of Sinus Rhythm

**DOI:** 10.3390/jcm10051029

**Published:** 2021-03-03

**Authors:** Amine El Amrani, Xavier Viñolas, Miguel Angel Arias, Victor Bazan, Pilar Valdovinos, Josep M. Alegret

**Affiliations:** 1 Department of Cardiology, Hospital Universitari de Sant Joan, IISPV, Universitat Rovira i Virgili, 43204 Reus, Spain; amin.elamrany@gmail.com (A.E.A.); mpilar.valdovinos@grupsagessa.com (P.V.); 2 Department of Cardiology, Hospital de la Sta. Creu i St. Pau, 08026 Barcelona, Spain; jvinolas@mac.com; 3 Department of Cardiology, Hospital Virgen de la Salud, 45004 Toledo, Spain; maapalomares@secardiologia.es; 4 Department of Cardiology, Hospital Universitari Germans Trias i Pujol, 08916 Badalona, Spain; victorbazang@yahoo.com

**Keywords:** atrial fibrillation, electrical cardioversion, pharmacological cardioversion, antiarrhythmic drugs

## Abstract

Background: Antiarrhythmic drugs (AADs) are frequently initiated in patients with persistent atrial fibrillation (AF) prior to electrical cardioversion (ECV), achieving pharmacological cardioversion (PCV) in some cases. Little is known about the mode of cardioversion and the effect of the type of AAD used in the maintenance of sinus rhythm (SR). Methods: From three national surveys of patients with persistent AF referred for ECV, we selected those who were pre-treated with AADs (amiodarone or group Ic AADs). We analyzed the effect of the type of cardioversion (pharmacological vs. electrical) and the AAD used in the maintenance of SR at three months. Results: Among the 665 patients selected, 151 had a successful PCV prior to the planned ECV. In the remaining 514 patients, 460 had a successful ECV. A successful PCV was related to a higher rate of SR maintenance than a successful ECV (77.9% vs. 57.5%; *p* < 0.0001). After a successful PCV, the maintenance of SR was identical in those patients treated with amiodarone and those treated with group Ic AADs (77.4% vs. 77.5%; *p* = 0.99), whereas after a successful ECV, amiodarone was clearly superior to group Ic AADs (61.3% vs. 43.0%; *p* = 0.001). Considering patients with successful PCV and ECV together, PCV was an independent factor related to the maintenance of SR. Conclusions: In patients with persistent AF, successful PCV selects a subgroup with a high probability of maintenance of SR. With regard to drugs, amiodarone was superior to group Ic AADs in patients with ECV, whereas in PCV, no differences were observed.

## 1. Introduction

In patient candidates for rhythm control strategies, electrical cardioversion (ECV) is the most commonly used method for restoration of sinus rhythm (SR) in patients with persistent atrial fibrillation (AF). This procedure is very effective in the acute setting, but recurrences are common [1,2,3]. With the aim of reducing recurrences after cardioversion, antiarrhythmic drugs (AADs) are frequently used prior to ECV and are often continued for a long period of time to avoid late recurrences [1,2,3,4]. Amiodarone appears to be the most effective drug for preventing recurrence [5,6,7], although at the cost of a higher incidence of long-term side effects, thus requiring individualized selection of AAD. In this context, AAD administered pre-ECV in patients with persistent AF has the potential to restore SR, inducing pharmacological cardioversion (PCV) in approximately 20–25% of patients before the planned ECV [3,4,5]. Many factors have been reported to be associated with the maintenance of SR after ECV [8,9,10], although there are few data about the influence of the mode of conversion (ECV or PCV) on the maintenance of SR. Moreover, there are no studies comparing the efficacy of different AADs after PCV in patients with persistent AF.

The aim of this study was to analyze, in patients with persistent AF treated with AAD pre-ECV, the influence of PCV prior to ECV on the maintenance of SR compared to ECV, as well as the effect of the type of AAD.

## 2. Materials and Methods

The REVERSE [11], CARDIOVERSE [12] and REVERCAT [13] studies featured surveys that prospectively recorded all patients with persistent AF who were considered candidates for ECV in 99 Spanish hospitals. These surveys sought to monitor clinical practice regarding ECV in Spain and were conducted by the same steering committee between 2003 and 2012 using common guidelines. The characteristics of the surveys have been described previously in detail [11,12,13]. The inclusion criteria were consistent between the three studies: ≥18 years of age, an AF duration ≥7 days, and no precipitating conditions such as hyperthyroidism, fever, recent thoracic surgery and pericarditis. The data recorded included the clinical characteristics, treatment details, echocardiography results and ECV procedure variables. The studies were conducted according to the principles of the Declaration of Helsinki and were approved by the Institutional Review Board and Ethics Committee of the Hospital Universitari de Sant Joan (1-12-04/10obs3, 10-02-25/2PROJ3, 11-12-22/12obs3). The participants provided informed written consent prior to participating in these studies.

Out of a total of 3263 candidates for ECV included in all 3 surveys, we considered those patients who were pre-treated with AAD and had a planned follow-up at 3 months. First, we selected those patients who had a successful PCV. From among the rest of the patients (who were treated with ECV), we selected those who underwent a successful ECV. For AADs, we considered only amiodarone and group Ic drugs (flecainide or propafenone) due to their capacity for restoring SR and the scarce number of patients treated with other drugs. Treatment with amiodarone was considered to have been used when it was initiated at least 3 weeks before ECV. Group Ic treatment was considered to have been used when it was started at least 48 h before ECV. A successful PCV was considered when reversion to SR was observed pre-ECV. Patients who did not cardiovert pharmacologically underwent ECV. Successful ECV was considered when SR was achieved, and the patient did not present immediate AF recurrence. AAD treatment had to have been maintained for at least 1 month after cardioversion for the patient to be selected for this analysis. At 3 months of follow-up, the number of patients in SR after PCV and ECV were analyzed, as well as were the factors involved in the maintenance of SR. The patient was considered to have remained in SR when SR was observed at the 3-month visit, and there was no evidence of recurrence of persistent AF during that period. Patients who underwent pulmonary vein ablation during follow-up were excluded (*n* = 10).

Structural heart disease was considered in the presence of any of the following anomalies: moderate or severe valvular heart disease; mitral stenosis of any grade; previous myocardial infarction; systolic dysfunction (EF < 50%); or any cardiomyopathy. Because of the date of the studies, the CHA2DS2VASC score was recorded only in the CARDIOVERSE study [12]. Patients could only be included once. In cases of recurrence, during the follow-up that required new cardioversion, the patient was not considered to have a new case.

Patients who, scheduled for ECV and without prior AAD treatment reverted to SR, were only registered in the CARDIOVERSE study. We also describe their number and follow-up.

### Statistical Analysis

Qualitative variables are expressed as percentages, and the differences were assessed using chi-square tests. Quantitative variables are presented as the mean ± standard deviation (SD), and the differences were evaluated using Student’s *t*-tests. A logistic multivariate regression analysis was performed to identify the independent variables related to the maintenance of SR at 3 months, focusing on the effect of the cardioversion mode and the type of DAA according to intention to treat. The variables included in the multivariate analysis were those that had a significance of *p* < 0.10 in the univariate analysis. Statistical significance was indicated by *p* < 0.05. All analyses were performed with IBM SPSS software (version 22).

## 3. Results

**Figure 1:**
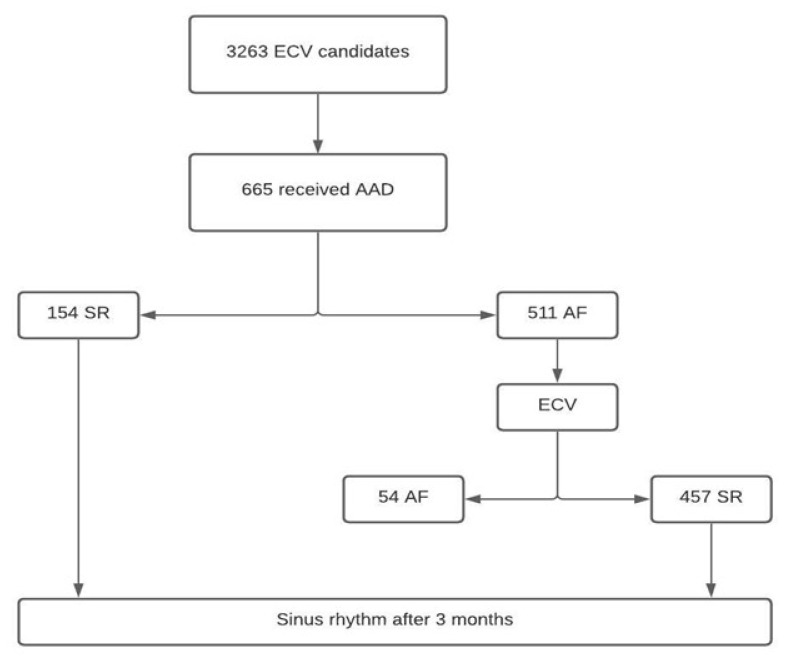
Selection process. AAD, antiarrhythmic drugs; AF, atrial fibrillation; ECV, electrical cardioversion; SR, sinus rhythm.

From among the patients included in the three surveys, we selected 665 patients (*n* = 211 from REVERSE, *n* = 180 from REVERCAT and *n* = 274 from CARDIOVERSE study, respectively) with persistent AF scheduled for ECV who were pre-treated with AADs and fulfilled the inclusion criteria. Of them, 154 patients (23.2%) reverted to SR, and the rest (511 patients) underwent ECV. Of these, 457 patients (89.4%) had a successful ECV, and 54 patients remained in AF. In summary, 611 patients were considered for the analysis (PCV *n* = 154; ECV *n* = 457). A flow chart is presented in Figure 1. The clinical characteristics of the patients are presented in Table 1. The mean age was 64 ± 10 years, and the patients were mostly male (70.5%). Patients with PCV had a better clinical profile, with a lower proportion of men, hypertension and history of previous ECV, lower body mass index and left atrial size and a higher left ventricular ejection fraction. Patients treated with amiodarone presented a higher proportion of structural heart disease and hypertension, were older, had a higher CHADS score and a lower left ventricular ejection fraction than patients treated with group Ic AADs (Supplementary Table S1).

**Figure 2:**
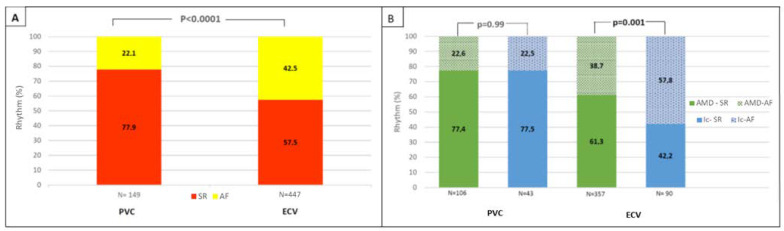
(**A**): Maintenance in sinus rhythm at 3 months according to cardioversion mode. (**B**): Maintenance of sinus rhythm at 3 months according to the antarrhythmic drug used. ECV, electrical cardioversion; PCV, pharmacological cardioversion; AMD, amiodarone; AF: atrial fibrillation; SR, sinus rhythm.

A high proportion of patients (*n* = 596; 98%; 97% from PCV group; 98% from ECV group) completed follow-up at three months and most of the patients (86%) maintained AAD treatment throughout the follow-up (86.6% in the PCV group, 85.9% in the ECV group). Of them, 373 patients (62.6%) remained in SR, with a higher rate observed for the PCV group (PCV 77.9% vs. ECV 57.5%; *p* < 0.0001) (Figure 2A). We observed different effects of AAD treatment on the maintenance of SR in the PCV and ECV groups (Figure 2B). Thus, in patients with successful ECV, amiodarone was superior to group Ic AADs (61.3% vs. 42.2%; *p* = 0.001), emerging as the only independent parameter associated with SR maintenance in the multivariate analysis (Supplementary Table S2). On the other hand, in patients with a successful PCV, the effect of amiodarone was identical to that of group Ic AADs (77.4% vs. 77.5%; *p* = 0.99). When we compared the effect of each AAD on PCV and ECV, both had higher protective effects in patients with a successful PCV (for amiodarone, 77.4% PCV vs. 61.3% ECV; *p* = 0.002; for group Ic AADs, 77.5% PCV vs. 42.2% ECV; *p* < 0.0001).

Considering patients with a successful PCV and ECV as a whole, we performed a logistic regression to analyze the factors related to maintenance of SR, with PCV remaining as an independent related variable (OR = 0.54 for recurrence [95% CI 0.33–0.89]) (Table 2).

Patients who, scheduled for ECV and without prior AAD treatment, reverted to SR, were only registered in the CARDIOVERSE study. In that study, 27 (5%) of the 530 patients without AAD treatment prior to ECV reverted to SR before the scheduled ECV. Of these, 26 were followed up at three months, and of these, 16 (61.5%) maintained SR.

## 4. Discussion

In this study, we found that in patients scheduled for ECV pre-treated with AAD, PCV was an independent predictor of the maintenance of SR. The major finding in this study was the different effect of the AADs in the maintenance of SR in patients with a successful PCV vs. ECV: in patients with ECV, amiodarone was superior to group Ic AADs, whereas in patients with PCV, the effect was identical.

Patients who reverted to SR with oral AADs prior to a scheduled ECV were more likely to be in SR at three months after cardioversion. This result was independent of the AAD administered. There is little evidence in the literature in this area. Oto et al. analyzed data from the Flec-SL-AFNET 3 study, a trial that assessed two strategies (short- and long-term treatment with flecainide) for the prevention of recurrence after ECV [14]. The patients included had persistent AF and received flecainide at least 48 h prior to the scheduled ECV. Using a data mining analysis, they identified PCV with flecainide as an independent predictor of maintenance of SR. On the other hand, in a small observational study, Galperin et al. analyzed the predictors of SR maintenance in patients with persistent AF who were pre-treated with amiodarone before ECV [15]. PCV during pre-treatment was one of the strongest factors predicting the long-term maintenance of SR. Our study adds new evidence from a large sample of patients using both AADs on the usefulness of PCV as a predictor of SR maintenance. None of the previous studies that studied the predictive value of PCV for maintaining SR analyzed the different effects of AAD on PCV and ECV. In our study, amiodarone was superior to the group Ic AADs in patients with successful ECV, even though the patients treated with amiodarone presented a worse clinical profile. On the other hand, in patients with a successful PCV, the effect of amiodarone was identical to that of group Ic AADs for the maintenance of SR. A hypothesis for this finding is that PCV could be a marker to identify patients with low complexity AF, enabling the selection of a subgroup of patients with higher probabilities of maintaining SR. Patients with PCV had a better clinical profile and, probably, less complex AF. Some studies suggest that the electrical complexity of AF is different among patients with persistent AF [14,16,17]. Both AADs could have high efficacy in the context of AF with a relatively better profile, which could be more sensitive to the effect of AADs. However, we should also consider that PCV could select a subgroup of patients with a high probability of maintaining SR, independent of the effect of the AAD.

It seems clear that successful ECV treatment with an AAD should be continued later with the purpose of reducing the incidence of recurrence [18]. In this sense, amiodarone seems to be the most effective AAD [1,3,5,18]. A recent meta-analysis emphasized the effectiveness of amiodarone for both restoration and maintenance of sinus rhythm after ECV for AF [6]. In our study, amiodarone was superior to the group Ic AADs in patients with successful ECV. The biggest disadvantage of amiodarone is its already known long-term side effects [19], which can cause its interruption in up to 20% of patients [20], and for this reason, decisions about which AAD is administered should be individualized. In the context of PCV, studies are lacking in reference to which AAD is more effective and the duration of the treatment. According to our results, some clinical consequences could be posed with respect to prophylactic antiarrhythmic treatment after successful PCV. Given the equality in efficacy, group Ic AADs seem to be the best option after a successful PCV, given the lower incidence of side effects. On the other hand, given the probable selection of a profile of patients with a high probability of maintaining sinus rhythm, regardless of antiarrhythmic treatment, it should be considered whether a short AAD course may be useful after PCV. Further studies should clarify these aspects.

Catheter ablation has become a cornerstone in the management of FA patients opting for a rhythm control strategy. In fact, recent studies propose that this treatment may be the first choice if this strategy is chosen [21,22]. Either way, ECV is a necessary treatment prior to ablation in patients with persistent FA. On the other hand, access to catheter ablation is highly variable throughout the world. Therefore, when making decisions about the indication for catheter ablation, it may be useful to know that the subgroup of patients that pharmacologically reverted to SR prior to CVD have a high probability of maintaining SR.

### Study Limitations

This study has the limitations of a retrospective analysis of a prospective observational study. Patients were not randomized to a specific drug, although a large number of patients included allowed subgroup comparisons in multivariate analysis. On the other hand, the lack of a control group prevents us from ensuring that the high rate of maintenance of SR after a PCV was due to the effect of AADs or if similar results could be achieved without AAD treatment after PCV. The potential effect of DAA could be due to two circumstances: PCV would select a subgroup of patients with an AF substrate more favorable to maintaining SR, or PCV could identify some patients especially sensitive to the effect of each AAD and, therefore, in which the prophylactic effect was more effective. We can only describe a small number of patients (*n* = 27; 5% of the total) from one of our surveys that reverted to SR prior to ECV without prior AAD treatment. These data supports the cardioverting capacity of AAD pre-ECV, but the small number patients prevents any assessment about SR maintenance.

## 5. Conclusions

Our study emphasizes the usefulness of PCV as a predictor of maintenance of SR. On the other hand, it adds new information about the behaviour of AADs in PCV and ECV for the maintenance of SR. Amiodarone was superior to group Ic AADs in patients with ECV, whereas in PCV, no differences were observed among AADs.

## Figures and Tables

**Table 1:** Baseline characteristics of the 611 patients included.

	N (%)	PCV	ECV	*p*
Age (years)	63.73 ± 10.29	63.36 ± 10.65	63.86 ± 10.18	0.602
Male gender	431 (70.5)	96 (62.3)	335 (73.3)	0.010
Previous heart disease	109 (17.8)	24 (15.6)	85 (18.6)	0.398
Myocardial infarction	21 (3.4)	4 (2.6)	17 (3.7)	0.508
Cardiomyopathy	43 (7)	8 (5.2)	35 (7.7)	0.301
Valvular heart disease	22 (3.1)	7 (4.5)	15 (3.3)	0.467
Others	23 (3.7)	5 (3.2)	18 (3.9)	0.696
Diabetes mellitus	90 (14.7)	16 (10.4)	74 (16.2)	0.079
Hypertension	335 (54.8)	71 (46.1)	264 (57.8)	0.012
COPD	57 (9.3)	15 (9.7)	42 (9.2)	0.839
LVH	148 (24,9)	32 (21.2)	116 (26.2)	0.221
ACEI or ARB	311 (50.9)	71 (46.1)	240 (52.5)	0.169
AF duration > 1 year	93 (15.2)	16 (10.4)	77 (16.8)	0.054
Previous CV	124 (20,4)	21 (13.6)	103 (22.6)	0.017
LA size > 50 mm	71 (12,6)	14(9.8)	57(13.6)	0.236
LA size (mm)	43.38 ± 5.74	41.53 ± 5.93	44.00 ± 5.54	0.000
LVEF (%)	58.61 ± 10.72	60.50 ± 10.38	57.95 ± 10.78	0.014
BMI (Kg/m^2^)	28.97 ± 4.48	27.52 ± 4.21	29.42 ± 4.43	0.000

ACEI, angiotensin-converting enzyme inhibitor; AF, atrial fibrillation; ARB, angiotensin receptor blockers; BMI, body mass index; COPD, chronic obstructive pulmonary disease; CV, cardioversion; ECV, electrical cardioversion; LA, left atrium; LVEF, left ventricular ejection fraction; LVH, left ventricle hypertrophy; PCV, pharmacological cardioversion.

**Table 2:** Parameters associated with sinus rhythm maintenance at 3 months in univariate and multivariate analysis.

	Univariate			Multivariate	
	SR	AF	*p*	OR (95% CI)	*p*
Age (years)	64.38 ± 10.46	63.08 ± 9.77	0.134		
Male gender	263 (70.5%)	158 (70.9%)	0.929		
Hypertension	202 (54.2%)	128 (57.4%)	0.441		
Diabetes mellitus	57 (15.3%)	32 (14.3%)	0.757		
Previous heart disease	72 (19.3%)	33 (14.8%)	0.162		
Myocardial infarction	13 (3.5%)	8 (3.6%)	0.979		
Cardiomyopathy	30 (8%)	12 (5.4)	0.173		
Valvular heart disease	14 (3.8%)	7 (3.1)	0.694		
Others	15 (4%)	6 (2.7%)	0.727		
COPD	40 (10.7%)	16 (7.2%)	0.151		
LVH	87 (23.7%)	56 (26.2%)	0.506		
ACEI or ARB	189 (50.7%)	114 (51.1%)	0.915		
AF duration > 1 year	48 (12.9%)	44 (19.7%)	0.025	0.671 (0.400–1.126)	0.131
Previous CV	63 (16.9%)	58 (26.2%)	0.006	0.639 (0.409–0.999)	0.050
LVEF	58.33 ± 11.13	58.96 ± 10.20	0.515		
LA size (mm)	43.01 ± 5.80	44.02 ± 5.54	0.048	0.979 (0.945–1.014)	0.237
LA size > 50 mm	44 (12.5%)	25 (12.6%)	0.956		
BMI (Kg/m^2^)	28.71 ± 4.70	29.49 ± 4.02	0.047	0.970 (0.930–1.013)	0.172
Maintenance of AAD	320 (85.7)	193 (86.5)	0.796		
PCV	116 (31.1)	33 (14.8)	0.000	0.543 (0.332–0.888)	0.015

ACEI, angiotensin-converting enzyme inhibitor; AF, atrial fibrillation; ARB, angiotensin receptor blockers; BMI, body mass index; COPD, chronic obstructive pulmonary disease; CV, cardioversion; LA, left atrium; LVEF, left ventricular ejection fraction; LVH, left ventricle hypertrophy; PCV, pharmacological cardioversion; SR, sinus rhythm.

## Data Availability

The data presented in this study are available on request from the corresponding author. The data are not publicly available due to privacy restrictions.

## Supplementary Materials

The following are available online at https://www.mdpi.com/2077-0383/10/5/1029/s1, Table S1: Baseline characteristics of the patients according to the antiarrhythmic drug used; Table S2: Parameters associated with sinus rhythm maintenance for ECV at 3 months in multivariate analysis.
