# Supplementary material for: Pharmacological Cardioversion after Pre-Treatment with Antiarrythmic Drugs Prior to Electrical Cardioversion in Persistent Atrial Fibrillation: Impact on Maintenance of Sinus Rhythm
Source: J Clin Med. 2021 Mar 3;10(5):1029. doi: 10.3390/jcm10051029 (PMC7958960; doi:10.3390/jcm10051029)
Supplement: Supplementary file 1 [file jcm-10-01029-s001.pdf]

Table S1. Baseline characteristics of the patients according to the antiarrhythmic drug used.

	Univariate		
	**Amiodarone**	**Ic group**	***p***
Age	64.29±10.28	61.56±10.09	0.008
Male gender	348 (71.6%)	83 (66.4,9%)	0.255
Hypertension	281 (57.8%)	54 (43.2%)	0.003
Diabetes mellitus	76 (15.6%)	14 (11.2%)	0.212
Previous heart disease	101 (20.8%)	8 (6.4%)	0.001
COPD	47 (9.7%)	10 (8.0%)	0.567
LVH	123 (26.1%)	25 (20.5%)	0.205
ACEI or ARB	260 (53.5%)	51 (40.8%)	0.011
AF duration > 1 year	68 (14.0%)	25 (20.0%)	0.095
Previous CV	94 (19.4%)	30 (24.2%)	0.235
LVEF	57.88±11.46	61.50±6.34	0.001
LA size	43.51±5.76	42.81±5.62	0.247
LA size > 50 mm	59 (13.1%)	12 (10.8%)	0.519
BMI	29.03±4.41	28.72±4.57	0.502
PCV	122 (25.1)	32 (25.6)	0.909

ACEI, angiotensin converting enzyme inhibitor; AF, atrial fibrillation; ARB, angiotensin receptor blockers; BMI, body mass index; COPD, chronic obstructive pulmonary disease; CV, cardioversion; LA, left atrium; LVEF, left ventricular ejection fraction; LVH, left ventricle hypertrophy; PCV, pharmacological cardioversion.

Table S2. Parameters associated with sinus rhythm maintenance for ECV at 3 months in multivariate analysis.

	**Multivariate**	
	**OR (95% CI)**	***p***
Age	1.019 (0.996-1.043)	0.102
Male gender	1.384 (0.814-2.353)	0.230
AF duration > 1 year	0.739 (0.420-1.299)	0.293
Previous CV	0.682 (0.413-0.1.127)	0.135
LVEF	0.987 (0.966-1.008)	0.228
LA size	0.985 (0.946-1.027)	0.482
BMI	0.969 (0.923-1.017)	0.200
Amiodarone pre-CV	1.957 (1.121-3.417)	0.018

AF, atrial fibrillation; BMI, body mass index; CV, cardioversion; ECV, electrical cardioversion; LA, left atrium; LVEF, left ventricular ejection fraction.
